# Allergic rhinitis and dental-supporting tissue diseases in children

**DOI:** 10.1097/MD.0000000000024780

**Published:** 2021-02-19

**Authors:** Wan-Yu Lai, Chang-Ching Wei, Lei Wan, Chen-Hao Mai, Cheng-Li Lin, Jeng-Dau Tsai

**Affiliations:** a Department of Chinese Medicine, China Medical University Hospital; b School of Chinese Medicine, China Medical University; c Children's Hospital, China Medical University Hospital; d School of Medicine, China Medical University, Taichung; e Department of Pediatrics, Chang Bing Show Chwan Memorial Hospital, Changhua County; f Management Office for Health Data, China Medical University Hospital; g Institute of Biostatistics, China Medical University; h School of Medicine, Chung Shan Medical University; i Department of Pediatrics, Chung Shan Medical University Hospital, Taichung, Taiwan.

**Keywords:** allergic rhinitis, children, cohort study, periodontal disease, pulpal and periapical disease

## Abstract

The etiology of dental-supporting tissue diseases in children is multi factorial and not merely related to oral hygiene. Therefore, in the present study, we investigated the relationship between children <18 years old with allergic rhinitis (AR) and the risk of dental-supporting tissue diseases.

Data from the National Health Insurance Research Database (NHIRD) of Taiwan were used to conduct a retrospective longitudinal cohort study. The study cohort comprised 378,160 patients with AR (AR group) and 378,160 patients without AR (non-AR group), who were selected through frequency matching based on age, sex, and the index year. The study patients were followed until dental-supporting tissue diseases occurrence, withdrawal from the National Health Insurance program, or December 31, 2013. Cox proportional hazards regression analysis was conducted to calculate the risk of dental-supporting tissue diseases in the AR group after adjustment for age, sex, and relative comorbidities.

The adjusted HRs of periodontal, pulp, and periapical diseases in AR children were higher than those in the non-AR controls (1.51, 95% CI: 1.50 to 1.53; 1.06, 95% CI: 1.05 to 1.07, respectively). The AR to non-AR HRs of these inflammatory dental diseases were particularly higher in children <6 years old and in boys. The HRs of periodontal, pulp, and periapical diseases were greatest in those with >5 AR-related medical visits/year (5.57, 95% CI: 5.50 to 5.56; 4.06, 95% CI: 4.00 to 4.12, respectively).

Children with AR had a greater risk of inflammatory dental-supporting tissue diseases, particularly those <6 years old with primary teeth, boys, and those with severe persistent AR.

**Bullet points**

- The present study is the first to utilize a large sample to investigate the association between childhood AR and inflammatory dental-supporting tissue diseases, including periodontal, pulp, and periapical diseases.
- Children with AR had a greater risk of inflammatory dental-supporting tissue diseases, particularly those < 6 years old with primary teeth, boys, and those with severe persistent AR.
- Identifying risk factors for inflammatory dental-supporting tissue diseases is important not only to prevent inflammation in the oral cavity but also to prevent multiple inflammation-related systemic diseases.

## 1 Introduction

Periodontal, pulp, and periapical diseases, inflammatory dental-supporting tissue diseases, are common dental disorders in children.^[1]^ The periodontal, pulp, and periapical tissues are closely associated with development, anatomy, and function. Pulp and periapical disease may affect the initiation and progression of periodontal disease.^[2]^ In contrast, patients with chronic periodontitis usually have pulpal tissue changes such as inflammation, edema, necrosis, fibrosis, and calcification.^[3]^ The exchange of immune cells and inflammatory mediators between periodontal, pulp, and periapical tissues is considered to happen through the vascular system in the apical foramen.^[2]^ Besides, a variety of systemic diseases are reported to interfere with periodontal, pulp, and periapical health.^[4,5]^

Chronic periodontitis and pulpitis may increase systemic inflammation, and are linked to multiple systemic diseases such as type 2 diabetes, metabolic disease, obesity, cardiovascular disease, fatty liver disease, and cancer.^[4,5]^ Therefore, identifying risk factors for periodontal, pulp, and periapical diseases in the pediatric population is important not only to prevent inflammation in the oral cavity but also to prevent multiple inflammation-related systemic diseases. However, despite increasing research on the link between dental-supporting tissue diseases and systemic diseases over the past few decades, the fundamental biological mechanisms of the association are not fully elucidated.^[4,5]^

A few studies show the relationship between periodontal disease or salivary micro-flora change and allergic disease.^[6,7]^. Allergic rhinitis (AR) is the most common allergic disease. Children with AR are frequently observed to have gingival and periapical inflammation.^[8,9]^ Interleukin (IL)-5,12,13, and IL-16, involved in the regulation of the onset and development of AR,^[10,11]^ also play a role in the pathogenesis of periodontal diseases.^[12]^ Several studies have discussed the relationship between AR and dental caries in children.^[8,13–15]^ Although some studies with inconsistent results have focused on adult patients^[13,15]^ there is no study demonstrating the relationship between AR and periodontal, pulp, and periapical diseases in childhood.

Because there are 3 stages, including primary, transition, and permanent, of teeth development in children, whether AR is an independent risk factor for dental-supporting tissue diseases in childhood is of great interest. This large, population-based study aimed to explore how AR correlates with childhood inflammatory dental-supporting tissue disease, including periodontal, pulp, and periapical diseases.

## 2 Material and methods

### 2.1 Data source

The National Health Insurance (NHI) program was established in Taiwan in 1995. This program has covered the reimbursement of medical care for approximately 23 million people, accounting for over 99% of the population of Taiwan.^[16]^ The NHIRD contains all the registry data of the insured, diagnostic codes of International Classification of Diseases, 9th Revision, Clinical Modification (ICD-9-CM), as well as details of outpatient and inpatient visits, procedures, prescriptions, and medical expenditure. This study used a dataset from the NHIRD, containing a randomly selected sample of half of all insured children in Taiwan. Based on the Personal Information Protection Act, de-identification was performed before the release of the dataset to researchers; thus, informed consent was not required for this study. This study received approval from the institutional review board of China Medical University Hospital (CRREC-103-048).

### 2.2 Study design and subjects

This was a retrospective cohort study using the NHIRD. Between 2000 and 2012, patients <18 years old with newly diagnosed AR (ICD-9 Clinical Modification (ICD-9-CM) code 477) were selected as the AR cohort. The comparison non-AR cohort with no AR diagnostic codes was selected by 1:1 matching based on a propensity score. The propensity score was calculated using a logistic regression model to estimate the probability of disease assignment based on baseline variables, which included age, sex, index year, urbanization, and the presence of the following comorbidities: chronic sinusitis (ICD-9-CM 473), obstructive sleep apnea (ICD-9-CM 327.23), asthma (ICD-9-CM 493), hypertrophy of the tonsils and adenoids (ICD-9-CM 474.1), and obesity (ICD-9-CM 278). Confounders such as age, sex, urbanization, and comorbidities were adjusted for in the analysis.

### 2.3 Confounders

Periodontal, pulp, and periapical diseases are multi factorial diseases. Well known risk factors for oral diseases include poor oral hygiene, frequent consumption of a sugary diet, female, and urbanization.^[1,17]^ Urbanization has been reported to be related to oral hygiene.^[18]^ The urbanization level, categorized by the population density of the residential area into 4 levels, with level 1 being the most urbanized and level 4 being the least urbanized, was adjusted in our study.

Obesity has been reported to be associated with periodontal disease^[19]^ and may be related to the frequent consumption of a sugary diet. There were no data on the frequency of sugary diet consumption. Therefore, obesity was used instead of frequency of sugar diet consumption. Chronic sinusitis and asthma are common comorbidities in children with AR. The etiology and medication, such as antibiotics and inhaled steroids, might be associated with oral disease.^[20,21]^ There is some evidence on the association between periodontal disease and obstructive sleep apnea.^[22]^ These comorbidities are thought to be confounding factors that increase the risk of periodontal, pulp, and periapical diseases.

Dental facial anomaly and children with disability could increase the risk of dental disease because of difficulty in maintaining dental health. Individuals with congenital anomalies (ICD-9-CM 740-759) and mental retardation (ICD-9-CM 317-319) were excluded. Individuals diagnosed with dental diseases and dentofacial anomalies before 2000 were also excluded.

### 2.4 Outcome measurement

The risk of periodontal, pulp, and periapical diseases was compared in the AR and non-AR cohorts between 2000 and 2013. All physician-diagnosed dental diseases were determined using diagnostic codes for periodontal (ICD-9-CM 523), pulp, and periapical diseases (ICD-9-CM 522).

### 2.5 Statistical analysis

Baseline characteristics of AR and non-AR cohorts were compared using standardized mean difference. Values of standardized mean differences ≤0.01 indicated a negligible difference in mean values between the AR and non-AR cohorts. We used a Cox proportional hazard regression model to compare the risk of periodontal, pulp, and periapical diseases in AR and non-AR cohorts, adjusted for age, sex, urbanization, and comorbidities. We also compared the risk of development of these diseases given the average frequency of AR-related medical visits. The cumulative incidence curves of periodontal, pulp, and periapical diseases in the study cohort were estimated using Kaplan-Meier analyses. All analyses were performed using SAS software, version 9.1 (SAS Institute, Cary, NC, USA), and a *P* value <.001 was considered statistically significant.

## 3 Results

A total of 378,160 AR patients were identified; 45.9% of these patients were girls. The mean (standard deviation, SD) age at AR diagnosis was 4.85 (3.82) years, and 73.6% were newly diagnosed before the age of 6 years (Table 1). More than half of the subjects with AR resided in urban areas (approximately 60.8%). There were no significant differences between the AR and non-AR cohorts in terms of age, urbanization (except the highest residential area), and comorbidity of chronic sinusitis, asthma, and obesity (Table 1).

**Table 1:** Demographics between children with and without allergic rhinitis (AR).

	Non-AR (N = 378160)	AR (N = 378160)	
	n (%)	n (%)	Standard difference
Age, years, mean (SD)^†^	4.87 (3.92)	4.85 (3.82)	0.005
Stratified age, years			
<6	273408 (72.3)	278206 (73.6)	0.03
6–11	75566 (20.0)	71291 (18.9)	0.03
≥ 12	29186 (7.72)	28663 (7.58)	0.005
Sex			
Girl	177481 (46.9)	173452 (45.9)	0.02
Boy	200679 (53.1)	204708 (54.1)	0.02
Urbanization^‡^			
1 (highest)	11378 (29.5)	113918 (30.1)	0.02
2	117316 (31.0)	110687 (30.7)	0.007
3	73769 (19.5)	73364 (19.4)	0.003
4 (lowest)	75697 (20.0)	74791 (19.8)	0.006
Comorbidity			
Chronic sinusitis	1675 (0.44)	1661 (0.44)	0.001
Obstructive sleep apnea	270 (0.06)	1383 (0.27)	0.05
Asthma	13149 (3.48)	13163 (3.48)	0.000
Obesity	287 (0.08)	256 (0.07)	0.003
Hypertrophy of tonsils and adenoids	484 (0.11)	1784 (0.35)	0.049

The analysis for the risk of periodontal, pulp, and periapical diseases in children without AR is shown in Table 2. Children with AR had a significantly higher risk to develop these inflammatory dental supporting tissue diseases, irrespective of differences in age, sex, or urbanization than those without AR. The adjusted hazard ratio (HR) was higher in the AR cohort than that in the non-AR cohort (1.51, 95% confidence interval: 1.50–1.53; 1.06, 95% confidence interval [CI]: 1.05–1.07) for periodontal disease and pulp and periapical disease, respectively. The ages <6, 6–11, and >11 years mark the 3 dentition stages primary, transitional, and permanent teeth stage, respectively. The risk of pulp and periapical diseases in the transitional teeth stage did not differ between the 2 cohorts; however, the risk for the 2 diseases was markedly higher in AR cohort than in the non-AR cohort for the other dental stages. The association between the annual frequency of medical visits due to AR and the risk of periodontal, pulp, and periapical diseases is shown in Table 3. The adjusted HR was higher for children with a high frequency of AR-related medical visits per year than for children without medical visits for AR. The dose-dependent relationship was found between a high frequency of AR-related medical visits and developing periodontal, pulp, and periapical diseases.

**Table 2:** The risk of periodontal disease, and pulp and periapical disease compared to children without allergic rhinitis (AR) stratified by demographics in Cox proportional hazard regression.

	Non- AR			AR			
	Event	Person-years	IR	Event	Person-years	IR	Adjusted HR^†^ (95% CI)
Periodontal disease							
All	56737	2717378	20.9	85054	2584093	32.9	1.51 (1.50, 1.53)^∗^
Sex							
Girl	28977	1215356	23.8	40614	1137300	35.7	1.48 (1.46, 1.50)^∗^
Boy	27759	1502022	18.5	44440	1446793	30.7	1.55 (1.53, 1.57)^∗^
Dentition stage^†^							
Primary	35604	2097124	17.0	55983	2032873	27.5	1.58 (1.56, 1.60)^∗^
Transitional	13527	532125	25.4	19008	468329	40.6	1.48 (1.45, 1.51)^∗^
Permanent	7605	88129	86.3	10063	82891	121.4	1.38 (1.34, 1.42)^∗^
Pulp and periapical disease							
All	60987	2594599	23.5	66223	2579405	25.7	1.06 (1.05, 1.07)^∗^
Sex							
Girl	27810	1172544	23.7	28990	28990	25.2	1.03 (1.02, 1.05)^∗^
Boy	33177	1422055	23.3	37223	37233	26.1	1.08 (1.07, 1.10)^∗^
Dentition stage^#^							
Primary	56421	1931383	29.2	61483	1952367	31.5	1.06 (1.05, 1.07)^∗^
Transitional	3143	567084	5.54	3302	531293	6.22	1.01 (0.97, 1.06)
Permanent	1423	96133	14.8	1438	95745	15.0	0.98 (0.91, 1.05)

**Table 3:** The risk of periodontal disease, and pulp and periapical disease among average frequency for medical visits of allergic rhinitis in Cox proportional hazard regression.

Average frequency for medical visit, per years	Event	Person-years	IR	Adjusted HR^†^ (95% CI)
Periodontal disease				
None	56736	2717378	20.9	1.00 (Reference)
≤3	42612	2094560	20.3	0.97 (0.95, 0.98)^∗^
4–5	9875	180652	54.7	2.78 (2.72, 2.84)^∗^
>5	32567	308881	105.4	5.57 (5.50, 5.65)^∗^
*P* for trend				<.001
Pulp and periapical disease				
None	60987	2594599	23.5	1.00 (Reference)
≤3	27587	2112652	13.1	0.57 (0.56, 0.58)^∗^
4–5	8272	178135	46.4	1.76 (1.72, 1.80)^∗^
>5	30364	288618	105.2	4.06 (4.00, 4.12)^∗^
*P* for trend				<.001

**Figure 1:**
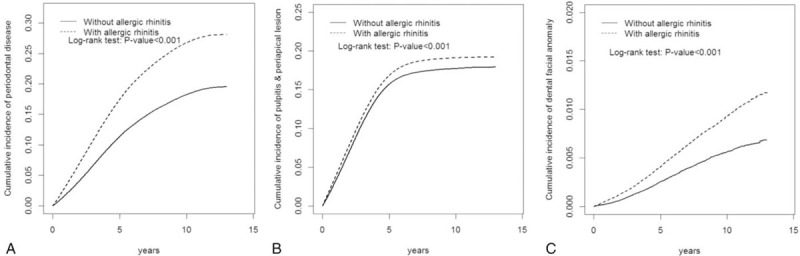
The Kaplan–Meier analysis of cumulative incidence of periodontal disease, and pulp and periapical disease for allergic rhinitis (AR) cohort compared to non-AR cohort.

Regarding the effect of comorbidities, a high HR for periodontal, pulp, and periapical diseases was noted even for AR children without comorbidities of chronic sinusitis, obstructive sleep apnea, asthma, and hypertrophy of tonsils and adenoids (Table 4). Figure 1 demonstrates the Kaplan–Meier analysis for the cumulative incidence of periodontal disease, pulp, and periapical diseases for the AR cohort compared to the non-AR cohort. (log-rank test, *P* < .001)

**Table 4:** The risk of periodontal disease, pulp and periapical disease among children with allergic rhinitis (AR) compared to children without AR stratified by comorbidity in Cox proportional hazard regression.

	Non- AR			AR			
	Event	Person-years	IR	Event	Person-years	IR	Adjusted HR^†^ (95% CI)
Periodontal disease							
Chronic sinusitis							
No	66145	3228924	20.5	105447	31.4	31.4	1.51 (1.50, 1.53)^∗∗^
Yes	302	12253	24.7	3477	9115	35.1	1.60 (1.43, 1.81)^∗∗^
Obstructive sleep apnea							
No	66397	3239850	20.5	108641	3446643	31.5	1.51 (1.50, 1.53)^∗∗^
Yes	50	1327	37.7	283	6525	43.4	1.12 (0.82, 1.51)
Asthma							
No	64970	3150083	20.6	87352	2666063	37.8	1.51 (1.49, 1.52)^∗∗^
Yes	1477	91094	16.2	21572	787105	27.4	1.68 (1.60, 1.77)^∗∗^
Hypertrophy of tonsils and adenoids							
No	66383	3237761	20.5	108559	3442232	31.5	1.51 (1.50, 1.53)^∗∗^
Yes	64	3417	18.7	365	10936	33.4	1.64 (1.25, 2.15)^∗∗^
Pulp and periapical disease							
Chronic sinusitis							
No	72843	3070794	23.7	84812	3335548	25.4	1.06 (1.05, 1.07)^∗∗^
Yes	244	12038	20.3	2286	101373	22.6	1.01 (0.88, 1.15)
Obstructive sleep apnea							
No	73058	3081474	23.7	86950	3430035	25.4	1.06 (1.05, 1.07)^∗∗^
Yes	29	1358	21.4	148	6886	21.5	1.17 (0.78, 1.76)
Asthma							
No	71034	2997545	23.7	66529	2670281	24.9	1.06 (1.05, 1.07)^∗∗^
Yes	2053	85287	24.1	20569	766640	26.8	1.09 (1.04, 1.14)^∗∗^
Hypertrophy of tonsils and adenoids							
No	73012	3079669	23.7	86853	3425832	25.4	1.06 (1.05, 1.07)^∗∗^
Yes	75	3163	23.7	245	11089	22.1	1.07 (0.82, 1.40)

## 4 Discussion

Chronic inflammatory diseases affecting dental tissues if not diagnosed promptly and treated appropriately may ultimately lead to tooth loss.^[1,2]^ Studies on the association between AR and inflammatory dental supporting tissue diseases have reported conflicting results.^[6,22,23]^ The present study is the first to utilize a large sample to investigate the association between AR and periodontal, pulp, and periapical diseases in children. We found that AR children had a significantly high risk and higher cumulative incidences of periodontal, apical, and periapical diseases than those without AR. A particularly high risk for these inflammatory dental supporting tissue diseases were found in those below age of 6 years with primary teeth and boys. Further, children with more frequent medical visits for AR had higher risks for periodontal, pulp, and periapical diseases, indicating a dose-dependent effect.

There are few studies investigating the interaction between AR and periodontal disease and the findings are contradictory. One cross-sectional study in Germany^[24]^ and the other one in Korea revealed an inverse association between periodontal disease and AR in adults. In contrast, another 2 matched case-control studies in Taiwan showed a positive correlation between AR and periodontal disease in adults.^[6,23]^ Moreover, Ho et al revealed that men, urban citizens, and low income people with AR had higher incidence of pulpitis.^[23]^ Our study found that AR children had a high risk of inflammatory dental supporting tissue diseases. Moreover, we also assessed the risks of these dental surrounding tissue diseases according to different stages of teeth development. Our results revealed that younger children with primary teeth had particularly higher risks for periodontal, pulp, and periapical diseases. Because chronic periodontitis and pulpitis may increase systemic inflammation and many systemic diseases, such as type 2 diabetes, metabolic disease, obesity, cardiovascular disease, fatty liver disease, and cancer, aggressive treatment of childhood AR may be beneficial for oral health improvement and for prevention of certain systemic diseases.^[4,5]^

Inhalation of corticosteroids has been reported to increase the incidence of caries and periodontitis because of a resultant change in oral pH, local deposition of steroids in the oral cavity, and their effect on oral mucosa1.^[4,23]^ However, there are limited studies on the association between intranasal steroids and dental inflammatory disease. The mainstay treatment of AR includes oral or intranasal antihistamines and intranasal corticosteroids.^[25]^ The management of AR depends on its severity and duration. Those with persistent and severe AR are at a higher risk of using intranasal steroids.^[25]^ We speculate that nasopharyngeal deposition of steroids due to postnasal drip might induce the same oral diseases that inhaled steroids do, which explains our finding that AR children with more frequent medical visits have a higher risk of periodontal, pulp, and periapical diseases.

Chronic polymicrobial infection to the surrounding dental tissues and eliciting a host inflammatory immune response in susceptible individuals is a central feature of periodontal, pulp, and periapical diseases.^[26]^ Our study showed that AR children had a higher incidence and risk of dental soft tissue inflammation. Although the pathogenesis remains unclear, dry mouth due to mouth breathing and taking an oral antihistamine and inflammatory reactions due to AR may worsen dental health.^[8,21]^ Mouth breathing may cause gingival surface dehydration, decreased epithelial resistance to bacterial plaques, and limit salivary auto-cleaning.^[27]^ Certain immunological and inflammatory reactions have been reported to interplay between AR and these dental diseases.^[11,12,28]^ Mast cells play a crucial role in the pathogenesis of AR.^[7]^ Several studies demonstrate that mast cells may also play an important role in the development of pulpitis and periodontitis during its acute stages and its subsequent transition to chronic inflammation.^[28]^ Histamine, released from mast cells, acts as a strong vasodilator and mediator of vascular permeability and may play a role in initiating pulp, periapical, and periodontal inflammation.^[29]^

There are several limitations of our study. First, the claims data do not include information on oral hygiene and the severity of periodontal, pulp, and periapical diseases. The urbanization used instead of oral hygiene due to association with oral hygiene behavior.^[18]^ Nonetheless, we report a positive and dose-dependent relationship between AR and development of periodontal, pulp, and periapical diseases. Second, the AR cohort was selected based on the diagnostic code of ICD-9-CM. Ideally, a skin prick test is necessary to confirm the AR diagnosis; however, the diagnoses for our study cohort were made by a licensed and well-trained physician.^[30]^ Third, surveillance bias should be taken into consideration for the AR cohort with a high risk of dental surrounding tissue diseases. Those with severe and persistent AR need more visits for medical care, which can trigger early screening for dental diseases. However, AR children with more medical care visits may have a higher chance to have oral health knowledge and surveillance, that may controvert this bias.

## 5 Conclusion

AR children had a higher risk for inflammation of dental supporting tissues, including periodontal, pulp, and periapical diseases. The risk was much higher in children aged less than 6 years with primary teeth, in boys, and in those with severe persistent AR. These findings highlight the importance of awareness and regular follow-up of dental conditions in AR children.

## Author contributions

∗Wan-Yu Lai and Chang-Ching Wei contributed equally.

Wan-Yu Lai, Chen-Hao Mai, and Chang-Ching Wei conceptualized and designed the study. Wan-Yu Lai and Chang-Ching Wei drafted the initial manuscript. Cheng-Li Lin carried out the acquisition of data and analysis and interpretation of data. Lei Wan and critically reviewed and revised the manuscript. Chang-Ching Wei and Jeng-Dau Tsai coordinated and supervised data collection, critically reviewed the manuscript, and approved the final manuscript as submitted.

**Conceptualization:** Chang-Ching Wei, Wan-Yu Lai.

**Data curation:** Wan-Yu Lai.

**Formal analysis:** Jeng-Dau Tsai.

**Funding acquisition:** Jeng-Dau Tsai.

**Investigation:** Chang-Ching Wei, Jeng-Dau Tsai, Lei Wan, Chen-Hao Mai.

**Methodology:** Jeng-Dau Tsai, Lei Wan, Chen-Hao Mai, Cheng-Li Lin.

**Resources:** Cheng-Li Lin.

**Software:** Chen-Hao Mai, Cheng-Li Lin.

**Supervision:** Chang-Ching Wei, Lei Wan.

**Validation:** Chen-Hao Mai.

**Writing – original draft:** Chang-Ching Wei.

**Writing – review & editing:** Chang-Ching Wei.
